# Increasing Compulsory Citizenship Behavior and Workload: Does Impression Management Matter?

**DOI:** 10.3389/fpsyg.2019.01726

**Published:** 2019-07-26

**Authors:** Fang Liu, Irene H. Chow, Man Huang

**Affiliations:** ^1^School of Management, Guangzhou University, Guangzhou, China; ^2^Department of Management, Hang Seng University of Hong Kong, Hong Kong, China

**Keywords:** impression management, ingratiation, exemplification, compulsory citizenship behavior, workload

## Abstract

The present study focuses on the dark side of impression management (IM) and proposes that IM tactics (ingratiation, exemplification, and their interaction) positively affect workload through the mechanism of compulsory citizenship behavior (CCB). We tested our hypotheses with data from 298 employees in China. Results revealed that ingratiation, exemplification, and their interaction, were positively related to workload, and CCB played a mediating role in all these relationships. We discussed the theoretical and practical implications of this study.

## Introduction

Impression management (IM) denotes the process in which individuals intend to affect others’ impressions of themselves (Rosenfeld et al., 1995). IM is a common phenomenon and a prominent social influence strategy to help or hinder individuals’ attempts to build, maintain, defend, or transform others’ impressions of themselves in organizational contexts. Individual-level IM empirical studies have predominantly focused on the bright side and positive results of IM for individuals, for example, higher organizational citizenship behavior ratings (e.g., Bolino and Turnley, 1999; Hui et al., 2000; Yun et al., 2007; Halbesleben et al., 2010; Chiaburu et al., 2015), greater performance ratings (e.g., Wayne and Liden, 1995; Bande et al., 2017), better assessments of interview performance (e.g., Roulin et al., 2014, 2015), and career success (e.g., Bolino et al., 2006). However, IM also has its dark side. It takes more time and effort to engage in proactive IM for individuals indeed. Even individuals’ authentic IM may have a negative impact on their colleagues’ their well-being (Turnley et al., 2013). Nevertheless, relatively few studies have attached importance to the dark side and negative results of IM for individuals.

A chief objective of our study focuses on the flip side of the coin, that is, investigates the dark side of IM. Drawing on the conservation of resources theory (COR theory; Hobfoll, 1989; Grandey and Cropanzano, 1999), if one’s resources are threatened or lost, (s)he is likely to feel stressed (Hobfoll, 1989). Hence, IM with increased energy (i.e., time and psychological resources) consumption, has a positive impact on workload. Workload is defined as the combination of work quantity and work pace (Karasek, 1985). It is important to note that workload is a desirable outcome in itself because it has negative impacts on a wide variety of work-related as well as nonwork-related outcomes, such as, turnover intention (Jones et al., 2007), psychological detachment (Sonnentag and Fritz, 2015), work-family conflict (Matthews et al., 2014; Goh et al., 2015), and life satisfaction (Goh et al., 2015).

We further argue that IM has a positive impact on workload through compulsory citizenship behavior (CCB). CCB refers to personal participation in extra-role activities that always go against one’s will, displaying a distinct dynamic different from voluntary beneficence (Vigoda-Gadot, 2006, 2007). Scholars have suggested that employees may display organizational citizenship behavior (OCB) for serving their private interest, including impressing the management (e.g., Yun et al., 2007; Chiaburu et al., 2015). However, few researchers have noted that OCB stemming from IM is an instrument actually and loses voluntary meaning. Managers do understand and emphasize the importance of OCB, although OCB is not officially approved in the formal incentive system, (Allen and Rush, 1998; Hui et al., 2000). Thus, we argue, more exactly, individuals tend to display CCB rather than OCB to fulfill unofficial assignments beyond the formal job responsibilities (Vigoda-Gadot, 2006). However, few researchers have paid enough attention to the impact of IM on CCB.

We also consider the interaction effects of different IM tactics. The majority of individual-level IM empirical research has examined IM tactics in isolation (e.g., Yun et al., 2007; Cheng et al., 2014), and seldom considered the use of different combinations of IM. However, IM tactics likely interact with each other (Bolino et al., 2008, 2016). Adopting ingratiation combined with exemplification is probably more useful than adopting ingratiation or exemplification separately. As Bolino et al. (2016) noted, there is a need for research to explore the use of multiple forms of IM tactics used in combination. We postulate that the interaction of ingratiation and exemplification has a positive effect on workload, and CCB mediates the relationship. Compared to engage in either ingratiation or exemplification, when employees engage in both high level of ingratiation and exemplification to impress others, they are likely to perform more CCB which draining more vital resources away from formal job responsibilities, thus leading to a higher level of workload on the basis of COR theory.

**FIGURE 1:**
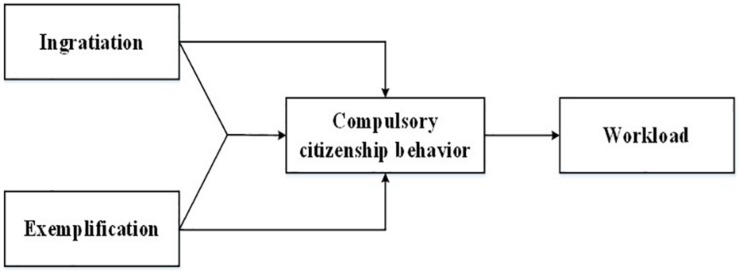
Theoretical framework.

The present study aims to make three important contributions. First, it investigates the negative effect of IM from a new angle. Drawing on COR theory, we demonstrate that IM (ingratiation, exemplification, and their interaction) exert a positive influence on workload by increasing energy (i.e., time and psychological resources) consumption. Second, it is among the first to provide theoretical and empirical accounts of CCB as a key mediating mechanism of the relationship between IM and its negative outcomes. Our results show that ingratiation, exemplification, and their interaction exert influence on workload through CCB. Third, by including both the single and interaction effect of ingratiation and exemplification, the current study provides a more integrated perspective of how IM tactics (i.e., ingratiation and exemplification) influences its outcomes. It is a response to a call for more attention to the interaction effects of different IM tactics (Bolino et al., 2008, 2016). Our results indicate that using ingratiation combined with exemplification is more significant than using ingratiation and exemplification in isolation to increase CCB and workload. Fourth, our data were obtained from a survey in China. Extant IM studies have been mainly launched based on Western cultural setting. However, IM and its effects are not exactly the same in a high-power distance and more collectivistic **cultural setting** (Xin and Tsui, 1996). Figure 1 depicts the overall theoretical model.

## Theoretical Background and Hypotheses

### Impression Management and Workload

IM denotes a process in which individuals intend to affect others’ impressions of themselves to attain a specific goal (Rosenfeld et al., 1995). Individuals always seek positive valued images and avert negatively images (Gardner and Martinko, 1988). IM tactics can be categorized as assertive/defensive and strategic/tactical (Tedeschi and Melburg, 1984). While defensive tactics are seen as passive and exploited to minimize or repair damage to one’s image, assertive tactics are viewed as proactive and used to create and boost a favorable image (Schlenker, 1980; Wayne and Liden, 1995; Lee et al., 1999). While strategic IM focuses on the long-term development through establishing prestige and positive image, including competence, responsibility, and leadership, tactical IM focuses on the detailed, short-term objectives (Lee et al., 1999). In the light of Rosenfeld et al. (1995) and Bolino and Turnley (1999), there are five categories of IM tactics: (1) ingratiation– including behaviors with the intent of seeking the attribution of likability from observers by doing favors or using flattery; (2) exemplification– containing behaviors with a goal of striving for eliciting the attribution of dedication from observers by self-sacrificing or going above and beyond the call of duty; (3) intimidation– involving behaviors with a purpose of appearing dangerous by signaling their power or potential to punish; (4) self-promotion– denoting behaviors with an aim of eliciting the attribution of competence and mastery by showing their abilities or accomplishments; and (5) supplication– comprising behaviors with an objective of appearing helpless and needy by advertising one’s own weakness or shortcomings. All five categories of IM tactics are tactical (short term)-assertive (proactive and initiated by the actor) forms of IM (Tedeschi and Melburg, 1984; Bolino et al., 2016). Ingratiation and exemplification are prosocial tactics directed at benefiting others (Jones and Pittman, 1982; Sosik et al., 2002), whereas intimidation, self-promotion, and supplication are more self-serving strategies directed at benefiting the self instead of others, even at the expense of others (Jones and Pittman, 1982; Sosik et al., 2002). In addition, ingratiation, exemplification, and self-promotion likely form favorable and positive impressions on others, whereas intimidation and supplication likely create unfavorable and negative impressions on others (Bolino et al., 2016). In accordance with our attention to CCB, this study focuses on two widely used prosocial tactics-ingratiation and exemplification-directed at benefiting others and forming a favorable and positive impression on others.

In the following, we argue that ingratiation and exemplification may associate with workload. Workload typically denotes the combination of work quantity and work pace (Karasek, 1985). Given his/her ability, workload means an individual’s cost to work at a specific performance level with specific requirements (Arellano et al., 2012, p.1790). Shirom et al. (2010) described workload as “the perception of having too many things to do or not having enough time to do the things one has to do”.

According to the COR theory (Hobfoll, 1989; Grandey and Cropanzano, 1999), an individual seeks to access and retain resources, including objects (e.g., food), personal characteristics (e.g., self-esteem), conditions (e.g., promotion), and energies (e.g., time). If one’s resources are threatened, or lost, (s)he is likely to feel stressed (Hobfoll, 1989). Workload can be regarded as a job demand or stressor involving energy (i.e., time and psychological resources) consumption. An individual likely invests extra resources if the job demand increases. We argue that either ingratiation or exemplification represents such an energy (i.e., time and psychological resources) consumption. Ingratiation or exemplification likely leads to reduced resources available to perform official duties due to limited resources. For example, the more time one spends on IM (including ingratiation and exemplification), the less time one has to fulfill job demands, and the conflict between the two domains is thus caused by inadequate resources to meet the needs of the two roles. Accordingly, resource drain likely arises resulting from a high level of ingratiation and exemplification, which results in a high level of workload. This may be due to inadequate time and effort required to complete formal duties (Edwards and Rothbard, 2000). Thus, we propose the following:

*H1a:* Ingratiation positively relates to the level of workload.
*H1b:* Exemplification positively relates to the level of workload.

### Impression Management and Compulsory Citizenship Behavior

IM facilitates in creating the desired image to gain intended results for self-promotion (Bolino and Turnley, 1999). Individuals engaging in IM aim to enhance their reputations as helpful, capable contributors in the eyes of their supervisor (Bolino et al., 2006). They also aim to earn higher levels of social status from their peers. IM would lead to some citizenship behaviors. Specifically, trying to impress others for larger compensation increases, recommendations for prestigious positions, and faster rates of promotion, an individual tends to devote himself/herself to engage in behaviors deemed desirable, beneficial, and valuable (Bolino et al., 2008). As noted by several scholars, image enhancement may be one reason to display OCB. For example, Bolino et al. (2006) showed that supervisor-focused IM tactics are positively related to OCB, and OCB serves as a mediator of the linkage between supervisor-focused IM tactics and supervisor evaluations of employee likability. Grant and Mayer (2009) indicated that high prosocial and IM motives predict a higher level of OCB directed to the organization (initiative) and coworkers (helping and courtesy). However, OCB was rated by supervisors rather than employees themselves in those studies. Those researchers have not noticed that because of pressure from the outside, OCB (e.g., altruistic behavior, conscientiousness, sportsmanship, and courtesy) resulting from IM loses the original voluntary nature (Vigoda-Gadot, 2006). As Hui et al. (2000) noted, OCB is instrumental for promotion, and an individual likely displays OCB before the promotion and declines in OCB after the promotion due to the instrumentality of OCB. Because OCB are often informally encouraged and rewarded in their organizations (Bolino et al., 2010), employees are likely eager to be regarded as “good soldiers” through IM tactics, but in fact, they are more likely to be “good actors”.

We further posit that to build, maintain, or improve his/her self-image by using ingratiation and exemplification, in fact, an individual might be more likely to engage in CCB than OCB. The occurrence of CCB originates from a re-examination of the concept of OCB (e.g., Vigoda-Gadot, 2006, 2007; Bolino et al., 2010). CCB emphasizes the more negative side of extra-role behavior (Porpora, 1989). Vigoda-Gadot (2006, 2007) described CCB as an individuals’ participation in extra-role activities against his /her will, displaying a distinct dynamic different from voluntary beneficence. Different from conventional OCB, CCB is “in fact anything but spontaneous behavior” (Vigoda-Gadot, 2006, p. 85). CCB emphasizes that an individual is forced to participate in some unofficial assignments beyond the formal job responsibilities (Vigoda-Gadot, 2006).

Ingratiation and exemplification seem to have a lot in common with CCB. Ingratiation, as a form of assertive IM, denotes that an individual strives to be likable through flattery and favor rendering (Tedeschi and Melburg, 1984; Bolino et al., 2016). For instance, an individual may participate in extra-role behavior to express friendliness, make a positive statement about coworkers’ performance, and praise his/her supervisor, respectively. Exemplification, as a form of assertive IM, denotes that an individual strives to show concentration or excellence by doing more or better than necessary (Tedeschi and Melburg, 1984; Bolino et al., 2016). It is commonly accepted that organizations value and favor an individual’s willingness to perform tasks beyond the official job duties (e.g., Katz and Kahn, 1978; Organ, 1988). Thus, to impress others by ingratiation or exemplification, especially immediate supervisors and powerful peers, an employee is likely to engage in informal and extra-role activities above and beyond the official job duties without any formal compensation, however, in face of social or managerial pressure. When (s)he refuses to engage in these activities, (s)he might not be able to improve his(her) self-image, and further have a detrimental impact on his(her) career prospects, such as, opportunities for advancement, organizational space, and social position (Vigoda-Gadot, 2006). In this sense, in order to gain future advantages or at least hold the current position, employees are motivated to impress others and engage in extra-role behavior proactively. Yet employees’ engagement in extra-role activities gradually becomes against their will (Vigoda-Gadot, 2007). In conclusion, employees likely take the initiative to adopt proactive ingratiation and exemplification and further engage in citizenship behaviors (they are “good actors” but not necessarily “good soldiers”), but this initiative is more likely to be against their true will. Consequently, we argue that a motivated individual who impresses others by ingratiation or by exemplification is likely to show CCB to enhance his/her self-image in the organization. Therefore, we hypothesize the following:

*H2a:* Ingratiation positively relates to compulsory citizenship behavior.
*H2b:* Exemplification positively relates to compulsory citizenship behavior.

### Compulsory Citizenship Behavior and Workload

Following the COR theory (Hobfoll, 1989; Grandey and Cropanzano, 1999), as noted earlier, CCB implies that employees have to invest cognitive, emotional, and physical resources in extra-role behavior and informal tasks beyond job duties against their free will (Vigoda-Gadot, 2006). Thus, fewer resources can be devoted to formal job responsibilities regarding limited resources. Specifically, once the definition of formal responsibilities is extended into the unofficial area of good intentions, the more resources an employee devotes to CCB, the fewer resources he or she spends at formal tasks. Therefore, resource drain can occur resulting from a high level of CCB similarly, which resulting in the heavier workload. Indeed, if they keep on fulfilling formal job demands at a high level, going beyond the scope of formal duties may make employees feeling drained, worn out, and depleted (Bolino et al., 2015). Bolino and Turnley (2005) found engaging in individual initiative positively relates to both job stress and role overload. Vigoda-Gadot (2007) showed that CCB positively relates to job stress and burnout. Thus, we propose the following:

*H3:* Compulsory citizenship behavior positively relates to workload.

### Interactive Effect of Ingratiation and Exemplification

Individuals typically use multiple IM tactics depleting different resources and involving multiple kinds of extra-role activities rather than a single IM tactic depleting the same resource and involving similar extra-role activities. However, the interactive effects of different IM tactics have received little attention (Bolino et al., 2008, 2016). The interactive effect of ingratiation and exemplification may be important. According to the COR theory (Hobfoll, 1989; Grandey and Cropanzano, 1999), compared to high ingratiation-low exemplification, low ingratiation-high exemplification, and low ingratiation-low exemplification, high ingratiation-high exemplification represents that employees deal with both tactics (i.e., ingratiation and exemplification) at the same time, or change from ingratiation to exemplification, or reversely from exemplification to ingratiation. All these activities need a higher level of energy (i.e., time and psychological resources) consumption. Conversion between two tactics involving different resources also leads to resource depletion. Thus, resource depletion will be multiplied. Ingratiation and exemplification with multiple resource depletion may reinforce each other with greater impact on workload. Accordingly, high ingratiation-high exemplification represents a higher level of energy (i.e., time and psychological resources) consumption, fewer resources available to fulfill job demands, and thus a higher level of workload. Similarly, dealing with both ingratiation and exemplification at the same time, employees need to engage in multiple rather than similar extra-role activities beyond the requirements of their formal job duties without any formal compensation. Ingratiation and exemplification involving multiple kinds of extra-role activities may reinforce each other with greater impact on CCB. Accordingly, if an employee is motivated to impress others through high ingratiation-high exemplification, (s)he is likely to perform at a higher level of CCB against his(her) will to improve the self-image at the workplace than other three ingratiation-exemplification combinations. Thus, we investigated the interaction effect of these two predictors. The combination of a high level of ingratiation and a high level of exemplification result in higher levels of CCB and workload. Therefore, it is hypothesized that:

*H4a:* Ingratiation and exemplification interact in the prediction of compulsory citizenship behavior with high ingratiation-high exemplification predicting higher compulsory citizenship behavior.
*H4b:* Ingratiation and exemplification interact in the prediction of workload with high ingratiation-high exemplification predicting higher workload.

### The Mediating Role of Compulsory Citizenship Behavior

Further, we argue that CCB may play a critical role in the IM-workload linkage. When employees engage in either ingratiation or exemplification to impress others for an organizational position and future advantage, they are likely to perform extra-role behavior involuntarily (i.e., CCB). Based on the COR theory (Hobfoll, 1989; Grandey and Cropanzano, 1999), CCB is likely to drain vital resources away from formal job responsibilities, thus leading to a high level of workload. In other words, the amount of workload that individuals experienced rests with the level of CCB caused by ingratiation or exemplification. Furthermore, when employees engage in both high level of ingratiation and exemplification to impress others, they are likely to perform more CCB which draining more vital resources away from formal job responsibilities, thus leading to a higher level of workload on the basis of COR theory. Hence, CCB likely mediates the ingratiation-workload relationship, the exemplification-workload relationship, and the interactive effect of ingratiation and exemplification on workload, respectively. Accordingly, it is hypothesized that:

*H5a:* Compulsory citizenship behavior mediates the effect of ingratiation on workload.
*H5b:* Compulsory citizenship behavior mediates the effect of exemplification on workload.
*H5c:* Compulsory citizenship behavior mediates the interactive effect of ingratiation and exemplification on workload.

## Materials and Methods

### Sample and Procedures

Consistent with previous studies (e.g., Vandenberghe and Panaccio, 2012; Butts et al., 2015; Ng and Yam, 2019), relying on the authors’ personal relations and professional networks, we recruited in total of 350 employees from diverse organizations to engage in our survey to achieve a heterogeneous sample of the working population. Our sample consisted of full-time employees from organizations across different industries in China. We randomly selected employees from different, gender, age groups, education level, job levels, and departments. First, employees were asked to provide their e-mail address to receive an invitation containing a link to an online questionnaire. We also introduced the objective of the research and ensured the confidentiality and anonymity in the e-mail. Then we sent them a reminder to complete the questionnaire after 10 days. Constructs, including ingratiation, exemplification, CCB, and workload, represents an individual’s internal states. Therefore, in this study, it may be logical to collect data from participants themselves using self-reported measurement methods. We distributed questionnaires to 350 recruits and received responses from 309 participants. There were 298 respondents offering complete and valid questionnaires, and the response rate was 85.1%. 58.4% of respondents were female. Respondents averaged 29 years of age and 14 years of education. They represented four managerial levels, namely non-manager (69.8%), junior manager (17.4%), middle manager (9.4%), and senior manager (3.4%).

### Measures

We translated and back-translated all scales from English to Chinese (Brislin, 1980). We measured all the items with 5-point Likert scales ranged from 1 to 5 (i.e., from strongly disagree to strongly agree).

#### Ingratiation and Exemplification

We measured ingratiation and exemplification using measures developed by Bolino and Turnley (1999). For ingratiation, an example item was “Compliment your colleagues so they will see you as likable”. For exemplification, an example item was “Stay at work late so people will know you are hard working”. Cronbach’s alphas for ingratiation and exemplification were 0.83 and 0.72, respectively.

#### Compulsory Citizenship Behavior

CCB was assessed by Vigoda-Gadot (2007)’s 5-item scale. An example item was “The management in this organization puts pressure on employees to engage in extra-role work activities beyond their formal job tasks”. Cronbach’s alphas for this scale was 0.74.

#### Workload

Workload was measured by Nystedt et al.’s (1999) three-item scale. A sample item was “It often happens that you have to work under great time pressure”. Its Cronbach’s alpha was 0.73.

#### Control Variables

We controlled for gender (0 = female, 1 = male), age, years of education, and managerial level (1 = non-manager, 2 = junior manager, 3 = middle manager, 4 = senior manager) to avoid possible confounding effects.

## Results

### Preliminary Analysis

Table 1 describes means, standard deviations, correlations, and reliabilities of the study variables. Ingratiation and exemplification were positively related to both CCB and workload (*r*s range between 0.172 and 0.305, *p*s < 0.01). CCB was positively related to workload (*r* = 0.320, *p* < 0.01). These results provided preliminary support for Hypotheses 1–3.

**TABLE 1:** Means, standard deviations, and correlations of the study variables.

	**Variables**	***M***	**SD**	**1**	**2**	**3**	**4**	**5**	**6**	**7**	**8**
1	Gender^*a*^	0.42	0.49	—							
2	Age	29.14	9.04	−0.091	—						
3	Education	14.06	2.67	0.248^∗∗^	−0.260^∗∗^	—					
4	Managerial level	1.46	0.80	0.005	0.268^∗∗^	0.040	—				
5	Ingratiation	3.70	0.62	−0.048	0.178^∗∗^	−0.121^∗^	0.127^∗^	0.83			
6	Exemplification	3.21	0.67	0.112	0.033	0.069	0.014	0.397^∗∗^	0.72		
7	CCB	3.23	0.67	0.062	0.012	0.024	0.074	0.230^∗∗^	0.305^∗∗^	0.74	
8	Workload	3.34	0.63	0.086	0.021	−0.021	0.068	0.172^∗∗^	0.202^∗∗^	0.320^∗∗^	0.73

*N = 298. Numbers 1–8 in the top row correspond to the variables in the respective sections of the table. Coefficient alpha values are presented in italics along the diagonal. ^∗∗^p < 0.01, ^∗^p < 0.05. ^a^ male = 1; Female = 0.*

To assess the construct validity of our measures of ingratiation, exemplification, CCB, and workload, we conducted the confirmatory factor analysis. The hypothesized four-factor structure fit the data well, χ^2^ (df = 95) = 277.1, *p* < 0.001, RMSEA = 0.08, NFI = 0.87, CFI = 0.92, IFI = 0.92, SRMR = 0.088. In addition, an alternative three-factor model (ingratiation and exemplification were combined as one factor) fit the data significantly worse, χ^2^ (df = 101) = 697.84, *p* < 0.001, RMSEA = 0.14, NFI = 0.72, CFI = 0.76, IFI = 0.77, SRMR = 0.12. These model comparison results showed that the measures did capture distinct constructs.

### Hypothesis Testing

Ingratiation and exemplification were centered to reduce possible multicollinearity. We used hierarchical regression analysis to test the positive effects of ingratiation and exemplification on workload and CCB as predicted by Hypotheses 1–2, the positive effect of CCB on workload as predicted by Hypothesis 3, the interactive effect of ingratiation and exemplification on CCB and workload as predicted by Hypothesis 4, and the mediating role of CCB as predicted by Hypothesis 5 (see Table 2).

**TABLE 2:** Results of hierarchical regression analyses.

	**The direct effects**				**The interactive effects**		**The mediating effects**	
**Dependent variables**	**CCB**		**Workload**		**CCB**	**Workload**	**Workload**	
**Model**	**M 1**	**M 2**	**M 3**	**M 4**	**M 5**	**M 6**	**M 7**	**M 8**
Gender	0.048	0.025	0.097	0.083	0.003	0.067	0.082	0.067
Age	0.031	−0.002	−0.002	−0.024	0.011	−0.015	−0.012	−0.018
Education	0.070	0.068	−0.049	−0.049	0.088	−0.035	−0.071	−0.058
Managerial level	0.013	0.000	0.070	0.060	0.004	0.063	0.066	0.062
Ingratiation		0.144^∗^		0.105^+^	0.177^∗∗^	0.128^∗^		0.082
Exemplification		0.240^∗∗∗^		0.154^∗^	0.214^∗∗∗^	0.136^∗^		0.081
Ingratiation × Exemplification					0.196^∗∗∗^	0.136^∗^		0.085
CCB							0.319^∗∗∗^	0.259^∗∗∗^
*R*^2^	0.009	0.113	0.014	0.061	0.149	0.079	0.115	0.136
Adjusted R^2^	−0.005	0.095	0.001	0.042	0.129	0.056	0.100	0.112
*F*	0.652	6.170^∗∗∗^	1.046	3.152^∗∗^	7.270^∗∗∗^	3.535^∗∗^	7.567^∗∗∗^	5.680^∗∗∗^

*N = 298; ^+^p < 0.1, ^∗^p < 0.05, ^∗∗^p < 0.01, ^∗∗∗^p < 0.001. The estimates are standardized coefficients.*

First, we entered four control variables. As indicated in Model 1, 3, All the four control variables had no significant effects on workload and CCB. In step 2, we examined Hypothesis 1 in Model 4 and Hypothesis 2 in Model 2. As expected, both ingratiation (β = 0.105, *p* < 0.1) and exemplification (β = 0.154, *p* < 0.05) were significantly related to workload as indicated in Model 4. Thus, Hypothesis 1 was supported, indicating that when individuals engage in more ingratiation and exemplification, they were likely to undertake a heavier workload. As shown in Model 2, both ingratiation (β = 0.144, *p* < 0.05) and exemplification (β = 0.240, *p* < 0.001) associated with CCB significantly. Hypothesis 2 thus was supported, indicating that when individuals engage in more ingratiation and exemplification, they were likely to perform more CCB. In step 3, Hypothesis 3 was examined in Model 7 with CCB entered. A significant positive effect of CCB on workload was found (β = 0.319, *p* < 0.001) as indicated in Model 7. Therefore, Hypothesis 3 was supported, indicating that when individuals perform more CCB, they were likely to undertake a heavier workload.

**FIGURE 2:**
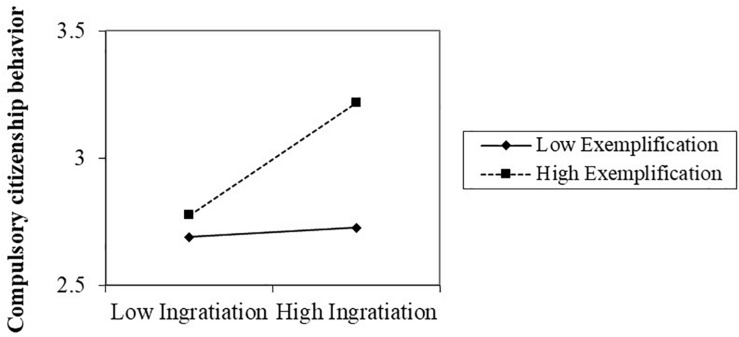
The interaction between ingratiation and exemplification on compulsory citizenship behavior.

**FIGURE 3:**
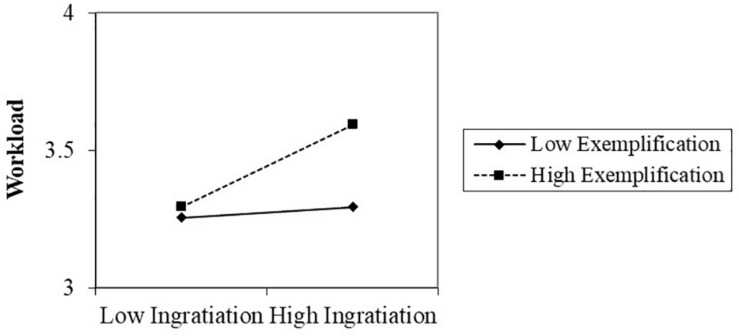
The interaction between ingratiation and exemplification on workload.

In step 4, we created an interaction term (ingratiation × exemplification) and added it in Model 5, 6, respectively. Ingratiation × exemplification associated with both CCB (β = 0.196, *p* < 0.001) and workload (β = 0.136, *p* < 0.05) significantly. Hypothesis 4 thus was supported, indicating that ingratiation and exemplification interact in the prediction of CCB as well as workload significantly. The interaction charts indicated that high ingratiation-high exemplification predicted higher CCB (see Figure 2) and heavier workload (see Figure 3) than the other three ingratiation-exemplification combinations. Therefore, Hypothesis 4 was supported.

Finally, based on Baron and Kenny’s (1986) approach, the first three requirements have been satisfied. Next, we judged whether the fourth requirement was met by entering CCB into Model 8. Compared to the positive ingratiation-workload relationship (β = 0.128, *p* < 0.05), the positive exemplification-workload relationship (β = 0.136, *p* < 0.05), and the positive ingratiation × exemplification-workload relationship (β = 0.136, *p* < 0.05) in model 6, after adding CCB to the model as shown in Model 8, CCB associated with workload positively (β = 0.259, *p* < 0.001), and the ingratiation-workload relationship, the exemplification-workload relationship, and the ingratiation × exemplification-workload relationship were no longer significant. These results suggested that CCB fully mediated the ingratiation-workload relationship, the exemplification-workload relationship, and the ingratiation × exemplification-workload relationship, respectively, providing support for Hypotheses 5.

To provide a more rigorous examination, we further performed the PROCESS Multiple Mediation Model 4 (Hayes, 2013). We set the Bootstrap sample size as 5,000, and control variables were included. Again, results indicated that a significant indirect effect occurred for ingratiation on workload through CCB, with a point estimate of 0.073 (*p* < 0.05) and a 95% confidence interval (CI) of [0.03, 0.14]. Further, exemplification had a significant indirect effect on workload through CCB, with a point estimate of 0.081 (*p* < 0. 05) and a 95% CI of [0.04, 0.14]. Finally, ingratiation × exemplification had a significant indirect effect on workload through CCB, with a point estimate of 0.067 (*p* < 0.05) and a 95% CI of [0.03, 0.13]. Therefore, these findings provided support for Hypothesis 5.

In addition, following the Podsakoff and Organ’s (1986) approach, we carried out Harman’s ex-post one-factor test, which loads all the items into an unrotated exploratory factor analysis to show whether a single factor accounts for the majority of the variance. Eigenvalues of four factors were larger than one. The largest one accounted for 26.992% of the variance. Hence, our findings could not be significantly affected by common method bias. Furthermore, all correlation coefficients were below 0.70, and all variance inflation factors were below 1.4, indicating that problems associated with multicollinearity are not serious (Neter et al., 1985; Tabachnick and Fidell, 2007).

## Overall Discussion

We discussed IM from a novel perspective and examined its potential negative outcomes. Drawing on the COR theory, we posited that both ingratiation and exemplification, as well as their interaction (ingratiation × exemplification), may have positive effects on workload, and CCB serves as an important mediator in those three relationships. Based on a sample of 298 employees, we confirmed the positive effects of both ingratiation and exemplification, as well as their interaction (ingratiation × exemplification) on workload. The results also provided support for the positive effects of both ingratiation, exemplification, and their interaction (ingratiation × exemplification) on CCB, and the positive effect of CCB on workload. We further found the mediating role of CCB in the ingratiation-workload relationship, the exemplification-workload relationship, and the ingratiation × exemplification-workload relationship, respectively.

### Theoretical Implications

First, we are absorbed in the reverse side of the coin and examine the dark side of IM at the individual level and show that IM (i.e., ingratiation, exemplification, and their interaction) can positively influence CCB and workload. As noted earlier, a large body of previous individual-level IM empirical studies has concentrated on the positive outcomes of IM, that is, higher OCB ratings (e.g., Bolino and Turnley, 1999; Hui et al., 2000; Yun et al., 2007; Halbesleben et al., 2010; Chiaburu et al., 2015), greater performance ratings (e.g., Wayne and Liden, 1995; Bande et al., 2017), higher assessments of interview performance (e.g., Roulin et al., 2014, 2015), and career success (e.g., Bolino et al., 2006). In other words, past research on IM at the individual level has primarily focused on the potential benefits of IM for employees, overlooking the potential harm that IM can cause employees. However, we argue that IM does not necessarily and always bring in benefits for employees. The results of this study imply that IM (ingratiation, exemplification, and their interaction) can result in CCB as well as workload, which substantially lower individual and even organizational performance.

Second, it is worth noting that workload is a recommendable outcome, since it has negative impacts on a wide variety of work-related as well as nonwork-related outcomes, such as, job satisfaction, organizational commitment, turnover intention (Jones et al., 2007), psychological detachment (Sonnentag and Fritz, 2015), work-family conflict (Matthews et al., 2014; Goh et al., 2015), and life satisfaction (Goh et al., 2015).

In addition, in response to the call that future studies should explore the influence of diverse IM tactics used in combination (Bolino et al., 2008, 2016), we empirically investigate the interactive effect of ingratiation and exemplification according to the COR theory. Previous IM research at the individual level has primarily been absorbed in the effects of IM tactics in isolation, ignoring interaction effects of different IM tactics. Results of the present study showed that ingratiation and exemplification are both significant predictors of CCB and workload and that there is an interactive effect of ingratiation and exemplification on both CCB and workload, respectively. These interactive effects indicate that beyond the main effect each predictor has on the criterion, there is a joint impact of the two variables on CCB and workload. More specifically, ingratiation seems to strengthen the positive effects of exemplification on both CCB and workload. Our finding that ingratiation and exemplification interact in the prediction of both CCB and workload with high ingratiation-high exemplification predicting higher CCB and heavier workload is important, as it expands our theoretical understanding effects of different IM tactics used in combination.

Another noteworthy implication is that this study furnishes a significant addition to the extant research by considering CCB as a mediating mechanism that links IM to its negative outcomes at the individual level. Our research is among the first to begin to unpack the black box between IM and its negative outcomes at the individual level. In the current study, we investigated how IM (ingratiation, exemplification, and their interaction) influences CCB, which further influences workload. In addition, our research reveals that IM (ingratiation, exemplification, and their interaction) translates into workload through increasing CCB. This is important because extant research has looked at the influence of IM on OCB (e.g., Bolino et al., 2006; Grant and Mayer, 2009), but little has noted that OCB originated from IM is instrumental and loses the voluntary meaning in reality (see Hui et al., 2000 as an exception). Following Hui et al. (2000), Vigoda-Gadot (2006), our research pays attention to the difference between OCB and CCB and identifies CCB as a mediator of the IM-workload relationship actually.

### Practical Implications

The current research also provides individuals and organizations with some important insights. IM has been considered to play a significant role in success at work and in life for individuals (Bolino et al., 2016). However, our findings indicate that ingratiation, exemplification and their interaction can have positive effects on both CCB and workload. In other words, IM does not necessarily imply benefits, and engaging in IM frequently, takes up too many resources, and finally results in CCB and workload. On the one hand, for individuals, unlike the general advice that suggest organizational members to take action to IM tactics that focus on either likability or competence, we suggest, to avoid the negative effects of IM, it would be beneficial for individuals to pay conscious attention to the frequency of IM, and only engage in authentic (rather than deceitful or fake) IM behaviors that targets and observers prefer (Leary, 1995).

On the other hand, from an organizational point of view, our findings suggest organizations and managers should develop a clearer awareness of the forms and the dark side of IM, especially for the interactive effects of different IM tactics, in order to ensure the accomplishment of the formal tasks. Although it can be a precious asset to appear likable and competent at times, such as, in sales talks or customer service, organizations and managers need to be aware that individuals’ sophisticated IM tactics may not only enhance personal impressions and even performance appraisals but also bring in CCB and workload. Managers should discourage employees from managing impressions too frequently, and create a better work environment where employees do not feel they have to engage in IM to be positively evaluated.

### Limitations and Future Research

First, although our study focused on the individual effects of employees’ IM tactics, it remains an interesting question of how employees’ IM tactics affect the performance of a group or the organization as a whole. It is possible that employees’ interaction with each other may influence the whole group’s performance and organizational performance eventually. Accordingly, the influences of individuals’ IM tactics at the individual level may be different from a higher level. In addition, team-level and organizational level IM should also be paid more attention.

Second, although we were interested in the negative effects of ingratiation and exemplification at the individual level, it is possible that the influences of diverse IM tactics are different from each other, thus, more work is still needed to comprehend the effects of other IM tactics, such as intimidation, self-promotion, supplication.

Third, reverse causality may be not just inferred from a cross-sectional design. For example, CCB may be antecedents of IM. Thus, we encourage future research to use longitudinal designs to make stronger inferences regarding the relationship among IM, CCB, and workload. Moreover, although it is logical to collect data by using self-reported measures from employees because ingratiation, exemplification, CCB, and workload all address individuals’ internal states, potential common method bias might arise because the variables were collected from the same source. Results of Harman’s ex-post one-factor test indicated that four factors larger than one appeared, and the greatest one interpreted 26.992% of the variance. In the present study, common method bias does not bring about serious consequences. In addition, the sample size of 298 respondents is a bit small. However, it should be better to collect multi-sourced and multi-time data and increase the sample size for future studies.

Fourth, we investigated how IM would affect CCB and workload by using an individual-based approach. Nevertheless, individuals are influenced by the social environment, and IM tactics might vary from culture to culture. For instance, compared to Caucasians, Asian managers is much more likely to apply tactics of flattering and exchange (Xin and Tsui, 1996). Our sample was from China, a high-power distance and more collectivistic cultural setting. To explore the generalizability of our findings, we call for more research looking at IM tactics and their dark side in more individualistic cultural backgrounds.

## Data Availability

The raw data supporting the conclusions of this manuscript will be made available by the authors, without undue reservation, to any qualified researcher.

## Ethics Statement

The present study was reviewed and approved by the Ethics Committee of Guangzhou University. All participants gave written informed consent in accordance with the protocol required by the Guangzhou University Human Subjects Protection Program to confirm their agreement to participate in the research.

## Author Contributions

FL and MH brainstormed about the research design and collected the data. FL analyzed the data and drafted the first manuscript. All the authors participated in the interpretation of the results and approved the final version and are jointly responsible for an appropriate review and discussion of all aspects included in the manuscript.

## Conflict of Interest Statement

The authors declare that the research was conducted in the absence of any commercial or financial relationships that could be construed as a potential conflict of interest.
